# Stem cell-derived small extracellular vesicles embedded into methacrylated hyaluronic acid wound dressings accelerate wound repair in a pressure model of diabetic ulcer

**DOI:** 10.1186/s12951-023-02202-9

**Published:** 2023-12-07

**Authors:** Letizia Ferroni, Ugo D’Amora, Chiara Gardin, Sara Leo, Luca Dalla Paola, Elena Tremoli, Alessandro Giuliani, Laura Calzà, Alfredo Ronca, Luigi Ambrosio, Barbara Zavan

**Affiliations:** 1 Maria Cecilia Hospital, GVM Care and Research, Cotignola, 48033 Italy; 2 Institute of Polymers, Composites and Biomaterials, National Research Council, Naples, 80125 Italy; 3 Department of Veterinary Medical Science (DIMEVET), University of Bologna, Ozzano Emilia, 40064 Italy; 4 Department of Pharmacy and Biotechnology and CIRI-SDV, University of Bologna, Bologna, 40126 Italy; 5 Translational Medicine Department, University of Ferrara, Ferrara, 44121 Italy

**Keywords:** Methacrylated hyaluronic acid, 3D bioprinting, Extracellular vesicle, Ulcer

## Abstract

Over the past years, the development of innovative smart wound dressings is revolutionizing wound care management and research. Specifically, in the treatment of diabetic foot wounds, three-dimensional (3D) bioprinted patches may enable personalized medicine therapies. In the present work, a methacrylated hyaluronic acid (MeHA) bioink is employed to manufacture 3D printed patches to deliver small extracellular vesicles (sEVs) obtained from human mesenchymal stem cells (MSC-sEVs). The production of sEVs is maximized culturing MSCs in bioreactor. A series of in vitro analyses are carried out to demonstrate the influence of MSC-sEVs on functions of dermal fibroblasts and endothelial cells, which are the primary functional cells in skin repair process. Results demonstrate that both cell populations are able to internalize MSC-sEVs and that the exposure to sEVs stimulates proliferation and migration. In vivo experiments in a well-established diabetic mouse model of pressure ulcer confirm the regenerative properties of MSC-sEVs. The MeHA patch enhances the effectiveness of sEVs by enabling controlled release of MSC-sEVs over 7 days, which improve wound epithelialization, angiogenesis and innervation. The overall findings highlight that MSC-sEVs loading in 3D printed biomaterials represents a powerful technique, which can improve the translational potential of parental stem cell in terms of regulatory and economic impact.

**Supplementary Information:**

The online version contains supplementary material available at 10.1186/s12951-023-02202-9.

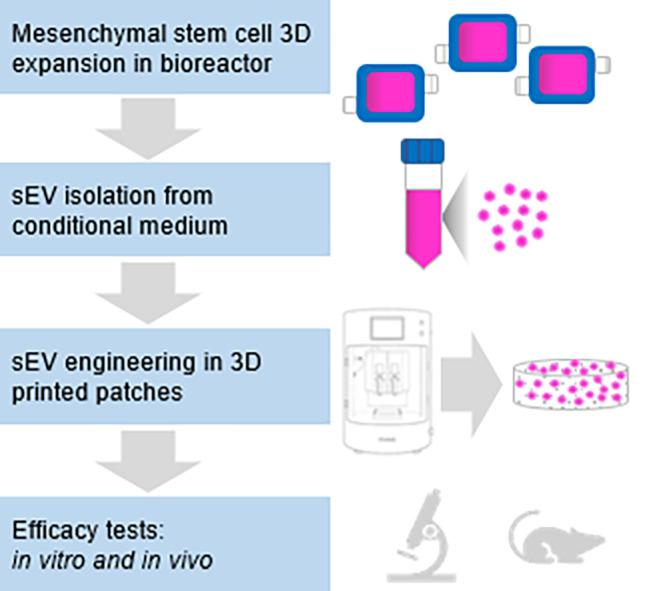

## Backgroung

In wound healing, different cell types precisely work together to coordinate the biological and molecular events of cell migration, proliferation, and extracellular matrix (ECM) remodeling [1]. Particularly, in chronic wounds, where the healing process may stall at various phases, the regular succession of events can be compromised. Indeed, these wounds are characterized by prolonged inflammation, ongoing infections, the development of antibiotic-resistant biofilms, and the inability of the local cells to react to cues that promote healing.

In the case of diabetic foot wounds, healing impairments are generally caused by neuropathy, ischemia, and trauma [2]. The conventional management of diabetic foot wounds comprises local surgical debridement, vascular assessment, treatment of active infections, application of wound dressings, and glycemic control [3]. A wide range of dressings is currently available for treating diabetic foot ulcers, depending on the lesion conditions (i.e., size, depth, extent of the wound) [4]. The fundamental function of a dressing is to create a moist environment at the wound interface that physiologically encourages cell migration and ECM production [3]. An ideal wound dressing should also allow gaseous exchange, be impermeable to microorganisms, and remove excess exudates. In addition, it should be non-toxic, non-allergenic, comfortable to the patient, non-adherent, easy to change and remove after healing, and be made from a sterile biomaterial [5, 6].

Hydrogels are frequently used as dressings for the treatment of skin wounds due to their unique combination of high water content, softness, flexibility, and biocompatibility [7]. They are also very attractive due to their tunable physico-chemical and biological properties [8]. However, they need to be suitably functionalized in order to improve their mechanical properties and their residence time [9]. To this aim, chemical or physical crosslinking are usually performed to obtain three-dimensional (3D) stable polymer networks [10]. Among natural polymers, hyaluronic acid sodium salt (HAs) has gained particular attention due to its similarity to the native ECM of several connective tissues. HAs is a glycosaminoglycan (GAG) involved in many tissues repair biological processes, including inflammation, angiogenesis, and ECM organization [11, 12].

Despite the many advantages of conventional wound dressings, these do not always result in reliably satisfactory outcomes. Consequently, there has been increasing interest in developing new advanced dressings for treating the compromised wound based on the patient’s specific conditions. In this context, 3D printing has recently emerged as an attractive option since it uses biocompatible inks for the creation of personalized dressings able to provide desired architectures [13]. These dressings can be functionalized with bioactive agents to generate the so-called bioactive wound dressings [14]. Apart from possessing most of the desirable characteristics of an ideal dressing, HAs, suitably functionalized, represents an interesting bioink for 3D printing because of its intrinsic rheological features [9, 15]. Furthermore, it is biodegradable, naturally lacks immunogenicity, and can be easily loaded with bioactive molecules [16–18].

In recent years, several studies have shown promising results in the management of chronic wounds by using hydrogels as carriers for the controlled delivery of small extracellular vesicles (sEVs) [19–21]. The sEVs, also called exosomes, are membrane vesicles, of 30–150 nm in diameter, secreted by most cell types in the extracellular space and containing a specific cargo, including DNA, RNA, proteins, and lipids, through which they mediate intercellular communications [22, 23]. Growing evidence has suggested that sEVs isolated from human mesenchymal stem cells (MSCs) could promote wound healing by modulating cellular function and enhancing angiogenesis [24, 25].

In this study, the main driving idea was to produce an advanced 3D wound dressing by 3D bioprinting using HAs derivative, as polymer matrix, and sEVs for the management of diabetic foot ulcers. To do this, the production of sEVs derived from human MSCs (MSC-sEVs) and their optimal working concentration was optimized through a series of in vitro experiments on human dermal fibroblasts and endothelial cells. Then, 3D printing technology was employed to manufacture methacrylated HA (MeHA)-based wound dressings, which were further functionalized with the MSC-sEVs. The biological efficacy of 3D printed MeHA dressings loaded with MSC-sEVs was then evaluated in diabetic mouse model of ulcer. Healing time, re-epithelization, angiogenesis, and re-innervation were finally evaluated.

## Results

### Production and characterization of MSC-sEVs

**Fig. 1:**
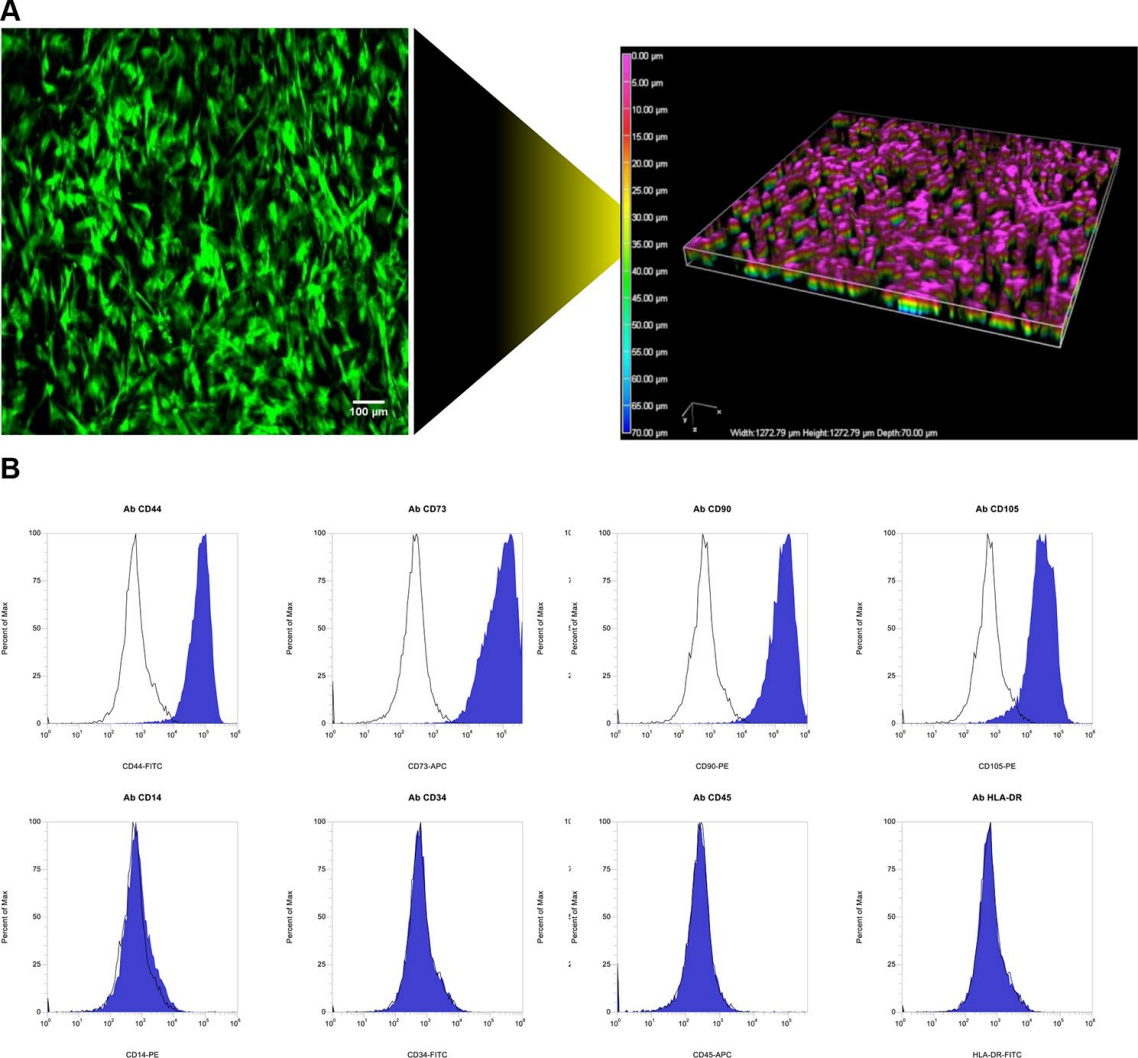
MSCs seeded and maintained in 3D bioreactor for up to 14 days. **(A)** Representative image of MSCs stained with Calcein-AM (green positivity) inside the bioreactor: maximum intensity projection of z-stack (left) and 3D view of z-stack confocal images (right). Scale bar 100 μm. **(B)** Pattern detection of MSC-specific surface markers by flow cytometry: positivity for CD44, CD73, CD90, and CD105 and negativity for CD14, CD34, CD45, and HLA-DR. MSCs (blue pick) and vehicle (black line)

Before sEV production, human MSCs were tested for stem cell multipotency [26]: they were adhered onto plastic plate under standard culture condition, and expressed the surface markers CD44, CD73, CD90, and CD105 (Figure S1). To increase cell expansion factor and reproduce a growth environment similar to physical condition, MSCs were seeded into handheld commercial 3D bioreactors [27]. The bioreactors were small system closed by a perimeter frame in continuity with two optical transparent membranes that allow oxygenation. Internally, a matrix of biocompatible polyester separated two chambers equipped with a port for input or output of medium, depending on liquid flow. Cells were injected with a syringe connected to input port, and retained by the inner 3D matrix. MSCs were maintained in bioreactors with EV-depleted medium up to 14 days. Every 2 days, fresh medium was injected in both chambers and conditioned medium (CM) was collected accordingly. After 14 days of 3D culture, cell viability was verified by calcein-AM staining, and stemness surface markers were tested by flow cytometry (Fig. 1). The probe calcein-AM permeates cell membranes in a non-fluorescent form, and then it is converted into green fluorescent derivative by the intracellular esterases. Therefore, the fluorescent signal revealed by confocal microscopy is proportional to cell growth and viability inside the bioreactor (Fig. 1A). Moreover, MSCs were positive for the surface markers CD44, CD73, CD90, and CD105 and negative for CD14, CD34, CD45, and HLA-DR (Fig. 1B), the specific surface marker profile of mesenchymal stem cells [26].

**Fig. 2:**
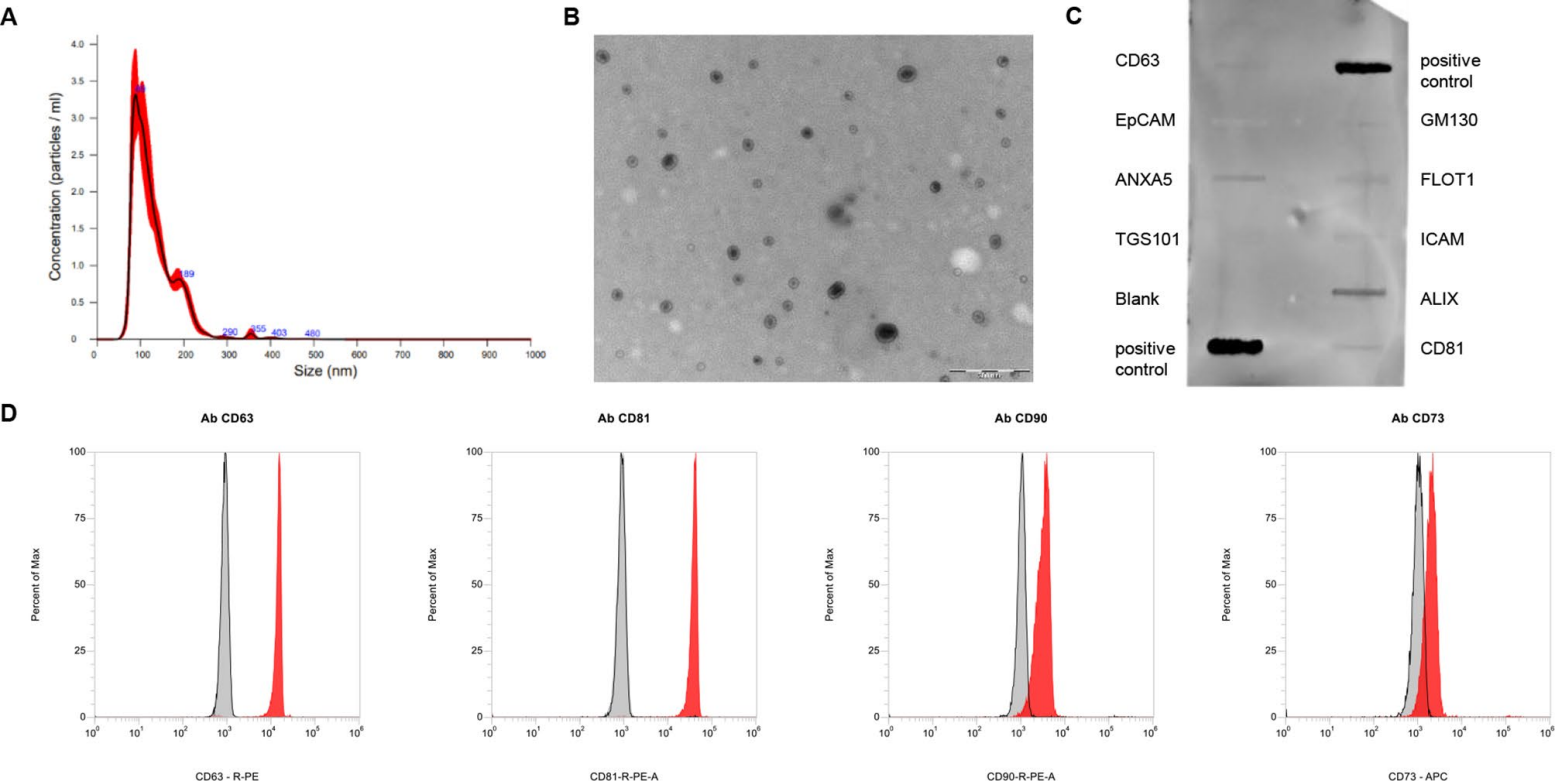
Characterization of MSC-sEVs. **(A)** NTA: distribution curve with a mean particle diameter of 130 nm. **(B)** Representative image of MSC-sEVs at TEM. **(C)** Western blot array for tetraspanin CD63, Epithelial Cell Adhesion Molecule (EpCAM), Annexin A5 (ANXA5), Tumor Susceptibility Gene 101 (TSG101), cis-Golgi matrix protein (GM130), Flotillin 1 (FLOT1), Intercellular Adhesion Molecule 1 (ICAM), programmed cell death 6 interacting protein (ALIX), and tetraspanin CD81. **(D)** Flow cytometry analysis: MSC-sEVs are positive for CD63 and CD81 exosome surface markers, and for CD90 and CD73 MSC specific surface markers. MSC-sEVs (red pick), EV-depleted medium (gray pick), and vehicle (PBS, black line)

The collected CM was firstly centrifuged to remove ultimate dead cells and cellular debris, and then EVs were isolated by ultrafiltration method. EVs were checked for dimension, morphology, and protein content. The nanoparticle tracking analysis (NTA) revealed a size distribution around 130 nm, corresponding to the typical diameter of small EVs. Precisely, particle diameter mean was 130 ± 1.7 nm and mode was 92 ± 3.9 nm. Particle size distribution by intensity showed a single peak ranging from 50 to 190 nm (Fig. 2A). Transmission electron microscopy (TEM) revealed round or oval membranous vesicle with nanometer dimension (Fig. 2B). A semi-quantitative exosome antibody array was performed to profile sEV proteins (Fig. 2C). Positive staining was observed for programmed cell death 6 interacting protein (ALIX), flotillin 1 (FLOT1), annexin A5 (ANXA5), and tetraspanins CD63 and CD81. A very faint signal was detected for intercellular adhesion molecule 1 (ICAM), tumor susceptibility gene 101 (TSG101), and epithelial cell adhesion molecule (EpCAM). Whereas, cis-Golgi matrix protein (GM130), the cellular protein contamination marker, was absent in sEV preparation. Moreover, flow cytometry analysis confirmed the sEV positivity for CD63 (96.1 ± 2.23) and CD81 (94.5 ± 4.51) along with the positivity for MSC-specific surface markers, CD90 (87.9 ± 5.72) and CD73 (58.1 ± 10.46) (Fig. 2D). The presence of such MSC specific surface antigens endorses the cellular origin of the sEV preparation [28]. Flow cytometry analysis was conducted comparing sEV preparation with bare EV vehicle (PBS) and bare EV-depleted medium; both controls gave negative results (Fig. 2D, black line and gray pick, respectively). All together these data showed that EVs isolated from human MSCs by ultrafiltration exhibited nanometer size below 200 nm, at least one protein from each category of transmembrane and intracellular proteins, as suggested by the Minimal Information for Studies of Extracellular Vesicles (MISEV) [29], and surface antigens specific of human MSCs, consequently the EV preparations can be defined as small EVs derived from human MSCs (MSC-sEVs).

### Biological effect of MSC-sEVs on recipient cells

A series of in vitro experiments were set up to identify the dose of MSC-sEVs able to positively influence the cellular functions of human endothelial cells and dermal fibroblasts, which are the prime functional cells of skin. In the first instance, the cellular uptake of the vesicles was investigated labeling MSC-sEVs with the green fluorescent dye PKH67. Then, to evaluate whether sEVs isolated from 3D-cultured MSCs can affect the biological properties of the recipient cells, increasing doses (20, 40, 80, 120 µg) of MSC-sEVs were incubated with cells for up to 48 h. The effect was evaluated in terms of proliferation, migration, and expression of specific markers; the functional role of sEVs on the angiogenic tube formation was additionally explored in endothelial cells.

**Fig. 3:**
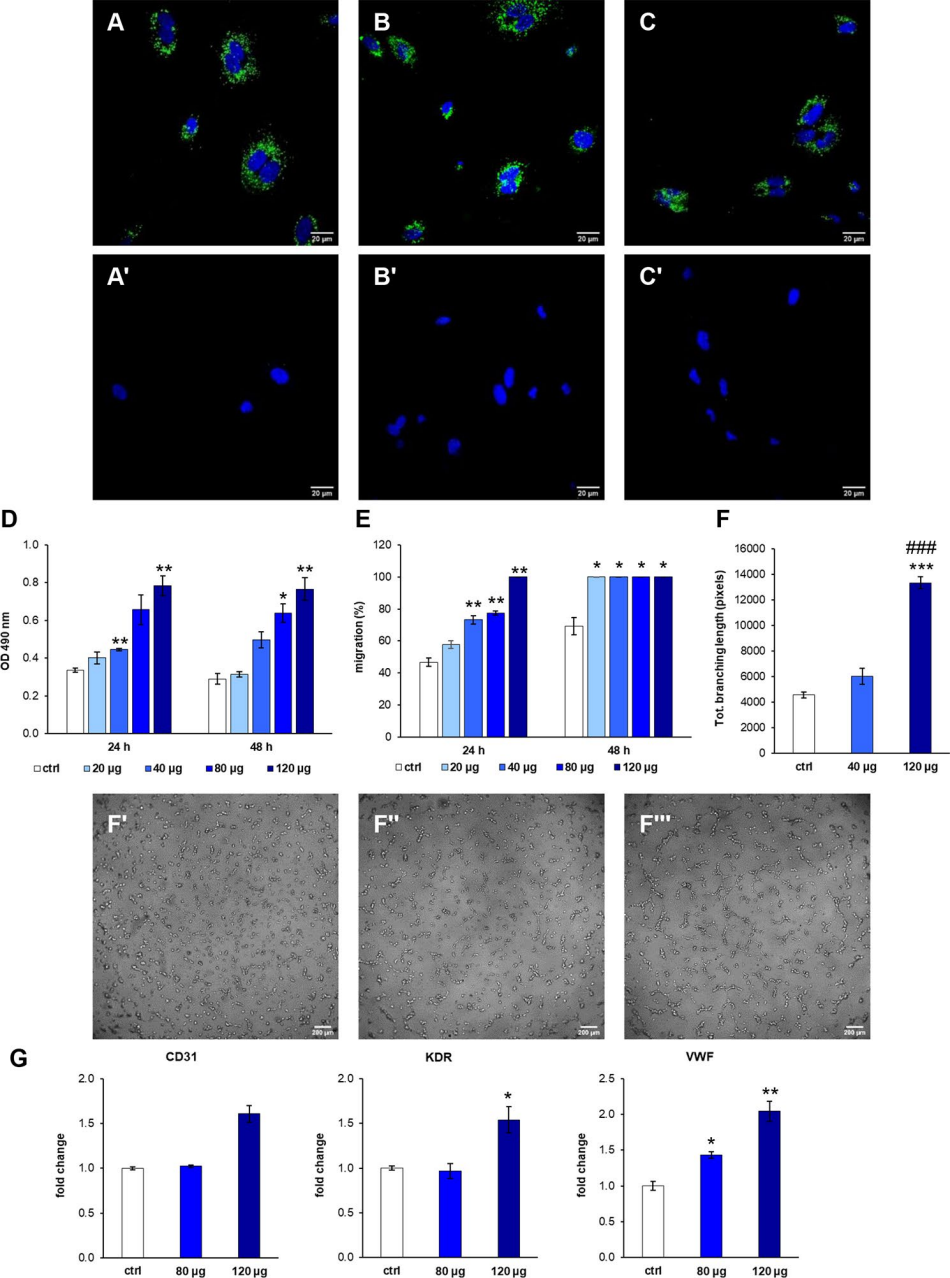
Treatment of human endothelial cells (HUVECs) with MSC-sEVs. Representative confocal microscopy images of the internalization of PKH67-labeled MSC-sEVs (green) in HUVECs (nuclei blue) after **(A)** 3 h, **(B)** 6 h, and **(C)** 24 h incubation. HUVECs incubated with PKH67-labeled PBS without MSC-EVs for (A’) 3 h, (B’) 6 h, and (C’) 24 h do not show any positivity to the probe. Scale bars 20 μm. **(D)** Proliferation and (E) migration of HUVECs treated with increasing doses of MSC-sEVs (20, 40, 80, 120 µg) compared to control (ctrl; no EVs). **(F)** Tube formation assay (corresponding total branching length, pixels, measured with the Fiji software) after 4 h incubation with 40 or 120 µg of MSC-sEVs and control. ***p < 0.001 compared to control; ###*p* < 0.01 compared to 40 µg. (F’-F’’’) Representative imagines after treatment with control, 40 µg, and 120 µg of MSC-sEVs, respectively. Scale bars 20 μm. **(G)** Gene expression of endothelial cell markers (CD31, KDR, and VWF) after 24 h incubation with MSC-sEVs. *p < 0.05, **p < 0.01, ***p < 0.001 compared to control

**Fig. 4:**
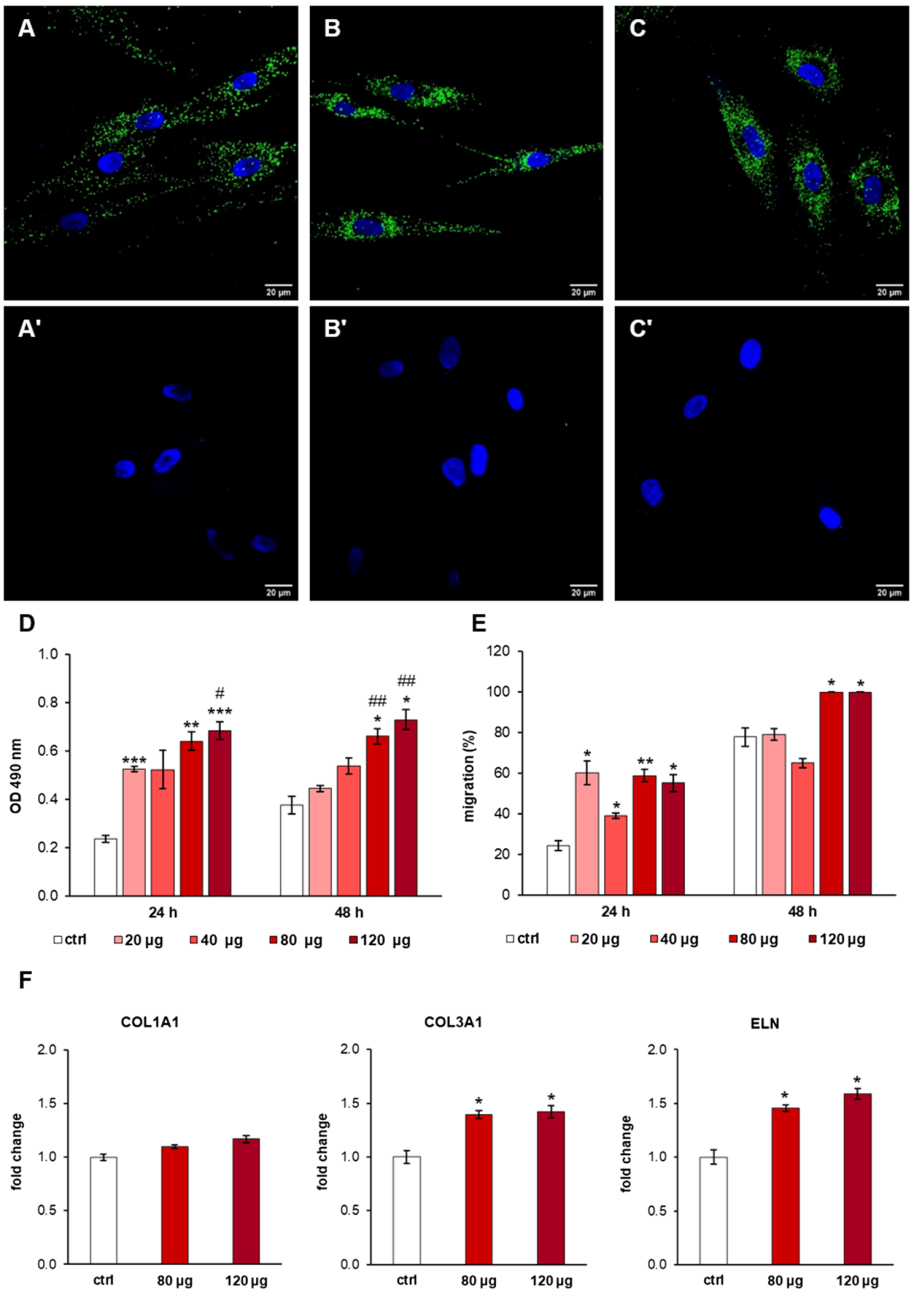
Treatment of human dermal fibroblasts with MSC-sEVs. Representative confocal microscopy images of the internalization of PKH67-labeled MSC-sEVs (green) in human fibroblasts (nuclei blue) after **(A)** 3 h, **(B)** 6 h, and **(C)** 24 h incubation. Fibroblasts incubated with PKH67-labeled PBS without MSC-EVs for (**A**’) 3 h, (**B**’) 6 h, and (**C**’) 24 h do not show any positivity to the probe. Scale bars 20 μm. **(D)** Proliferation and **(E)** migration of fibroblasts treated with increasing doses of MSC-sEVs (20, 40, 80, 120 µg) compared to control (ctrl; no EVs). **(F)** Gene expression of endothelial cell markers (COL1A1, COL3A1, ELN) after 24 h incubation with MSC-sEVs. *p < 0.05, **p < 0.01, ***p < 0.001 compared to control (0 µg)

For cellular uptake study, PKH67-labeled MSC-sEVs or control (PKH67-labeled vehicle) were incubated with cells up to 24 h and the internalization was observed under the confocal laser microscopy (Fig. 3A-C’, 4 A-C’). Microscopic observation revealed the fusion of the sEV membranes, visible as green fluorescent spots, with the membrane of recipient cells at 3, 6 and 24 h post incubation (Fig. 3A-C’, 4 A-C’). In both endothelial cells and dermal fibroblasts, MSC-sEVs were distributed throughout the cell surface at 3 h (Fig. 3A A); in contrast, they were mainly concentrated in the perinuclear region after 6 (Figs. 3B and 4B) and 24 h (Fig. 3C and D). No green fluorescence was observed in the control condition at tested time points (Fig. 3A’-C’, 4 A’-C’), confirming that the fluorescent spots were representative of the fusion of the fluorescent sEVs with the membrane of target cells.

The treatment with MSC-sEVs stimulated the proliferation and migration of endothelial cells (human umbilical vein endothelial cells, HUVECs) at both 24 and 48 h post-stimulation (Fig. 3D and E). Compared to the untreated cells, HUVECs treated with 120 µg of MSC-sEVS showed significant (*p* < 0.01) increase in proliferation both at 24 and 48 h (Fig. 3D). Instead, migration was significantly enhanced for treatment above 40 µg (Fig. 3E). Additionally, MSC-sEVs positively affected the tube formation in HUVEC culture (Fig. 3F and F’’’). Cells were seeded on Matrigel® and cultured with (Fig. 3F’’-3F’’’) or without (Fig. 3F’) MSC-sEVs to measure total branching length as an indication of the ability of HUVECs to form tubes. MSC-sEVs enhanced tube formation compared to control cells, and sEVs at the highest concentration (120 µg *p* < 0.001) showed the greatest total branching length as early as 4 h (Fig. 3F). Moreover, the gene expression profile of the endothelial markers CD31, kinase insert domain receptor (KDR), and von Willebrand factor (VWF) was investigated (Fig. 3G). Treatment with 120 µg of MSC-sEV resulted in a general increase in gene expression that is significant (*p* < 0.05) for KDR and vWF. In human dermal fibroblasts (HDF), MSC-sEVs promoted the proliferation and migration in a dose-dependent manner at both 24 and 48 h. Compared to the control group, MSC-sEV doses greater than 80 µg significantly increased proliferation and closed the scratch in wound assay after 48 h (Fig. 4D and E). Gene expression studies revealed no significant difference in the expression of collagen type I alpha 1 chain (COL1A1) between the sEV-treated and control groups (Fig. 4F, left panel). On the contrary, mRNA expression levels of collagen type III alpha 1 chain (COL3A1) (Fig. 4F, middle panel) and elastin (ELN) (Fig. 4F, right panel) were significantly (*p* < 0.05) increased, and the effect was evident at both 80 and 120 µg.

### MeHA patches development and MSC-sEVs loading

**Fig. 5:**
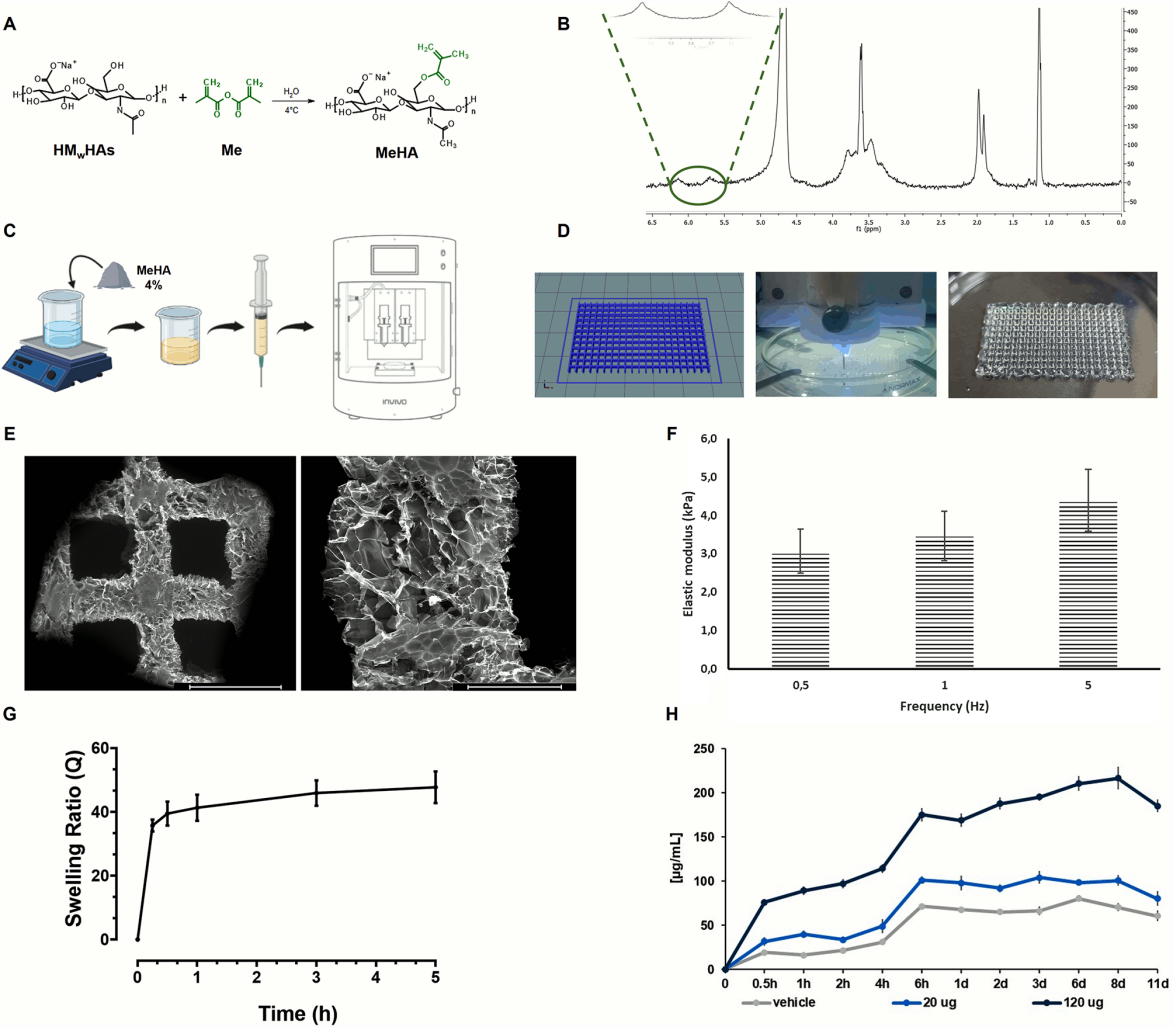
Schematic representation from the synthesis of the bioink to the characterization of the 3D printed patches. **(A)** Reaction scheme between HM_w_HAs and Me to produce MeHA. **(B)**^1^ H NMR spectra of MeHA. **(C)** 3D printing of MeHA. **(D)** From left to right: G-code, printing process by using UV light and picture of 3D printed patch, respectively. **(E)** SEM images of 3D printed patch. Scale Bar: 2 mm (left) and 500 μm (right). **(F)** Results obtained from DMA tests performed on 3D printed patches. **(G)** Swelling behavior of MeHA patches up to 5 h. **(H)** MSC-sEVs release from MeHA patches from 30 min to 11 days

The physicochemical and biological properties and the close similarity to the ECM of HAs has made it the ideal candidate as biomaterial for tissue engineering applications [9]. However, the small molecular weight fragments, as byproducts of the degradation process, showed pro-inflammatory effects of HAs with low molecular weight (below 340 kDa) [30]. In this work, high molecular weight hyaluronic acid (HMwHAs) was employed due to its biological and anti-inflammatory properties. Although HMwHAs has been reported to be used for bone tissue engineering [31, 32], its viscoelastic properties limit the application in 3D bioprinting. In this scenario, methacrylation was adopted to yield photo-crosslinkable HAs with increased mechanical stiffness and long-term stability and degradation time, without altering its cytocompatibility (Fig. 5A). Indeed, methacrylation allows hydrogels to be printable because the patches can be crosslinked with UV light during printing to fix their shape. Moreover, by improving the degradation time of the patch a more controlled release of the EVs can be obtained. To assess the presence of methacrylic moieties on the HAs backbone, ^1^ H NMR was performed (Fig. 5B). The ^1^ H NMR spectra showed typical peaks of HAs at 1.9 ppm related to the proton of the methyl (-CH_3_) group, while the protons from the D-glucuronic and N-acetyl glucosamine units were responsible for the peaks in the range of 3.3–5.6 ppm. Additionally, two peaks at 5.6 and 6.1 ppm were observed, and these peaks were associated with the added methacrylate moieties. 3D porous patches, measuring 50 × 50 × 3 mm^3^, were realized by “Rokit Invivo 4D2” (Fig. 5C) and cut into smaller pieces for the following characterization. It is widely known from literature that hydrogel showed partial collapse of the fiber while printing and for this reason scaffolds are always printed with low infill value to avoid fibers coalescence while printing [32]. An accurate study about the patches design optimization has been reported in the supporting information. Different infills have been tested from 40 to 70% to find the best balance in terms of pore dimension and interconnection. The 40% structure showed an open pore structure with pores too large to optimize the cell seeding (Figure S2 a). Increasing the infill to 60% the porosity starts to be closed due to the fibers collapse and the same could be seen for the 70% infill structure (Figure S2 c,d). For the 50%, instead, we have the best compromise in terms of pore diameter and interconnection. In this scenario, structures with 50% infill showed an open porosity that allow cell seeding, nutrient exchange, wound draining and transpiration (Figure S2 b).

MeHA patches were thoroughly analysed from a morphological and mechanical perspective (Fig. 5E F). The porous microstructure of the MeHA patches was evaluated in terms of size and shape by examining the overall structure with a Nikon digital camera (Fig. 5D) and by SEM (Fig. 5E). As clearly observed, MeHA maintained their lattice-like structure with uniform pore distribution, also after the freeze-drying process (Fig. 5E). Particularly, the pores displayed a size of 1.34 ± 0.06 mm, in dry state. Additionally, as can be seen, the patches displayed an interconnected macroporosity in accordance with the CAD design (Fig. 5D, left).

Dynamic mechanical analysis (DMA) was employed to assess the mechanical properties of 3D printed MeHA patches reported in Fig. 5F. MeHA patches showed a value of E’ ranging from 3.02 ± 0.57 kPa (0.5 Hz) to 4.39 ± 0.80 kPa (5 Hz) that is in agreement with those reported in the literature by Poldervaart et al. [32]. The 3D printed patch must be able to absorb MSC-sEVs and release them in situ. MeHA patches showed a steady swelling behavior (Fig. 5G). Particularly, dried patches were able to swell up to a value of 4000% within the first 30 min before achieving equilibrium within 1 h after immersion. The overall findings suggested that MeHA patches were suitable for wound healing applications since they significantly swell in physiological solution while preserving their 3D lattice-like structure throughout time. Regarding the MeHA patch stability, we tested the structure stability till 15 days and no degradation has been detected. The results of the degradation test have been reported in the supplementary information (Figure S3) and the structure appeared to be stable till day 15 with no weight change detected.

The 3D printed MeHA patches were loaded with MSC-sEVs, as previously described elsewhere [18]. To limit the exposure to chemical compounds for hydrogel synthesis and printing, two doses of sEVs (20 and 120 µg) were loaded in the MeHA patches post-printing. The release of MSC-sEVs from MeHA patches was evaluated by incubation in serum-free culture medium at 37 ◦C for up to 11 days. The quantification with the BCA assay showed that EV-release from patches was proportional to the previously loaded doses (Fig. 5H).

### MSC-sEVs and MeHA patches application on pressure ulcer animal model

**Fig. 6:**
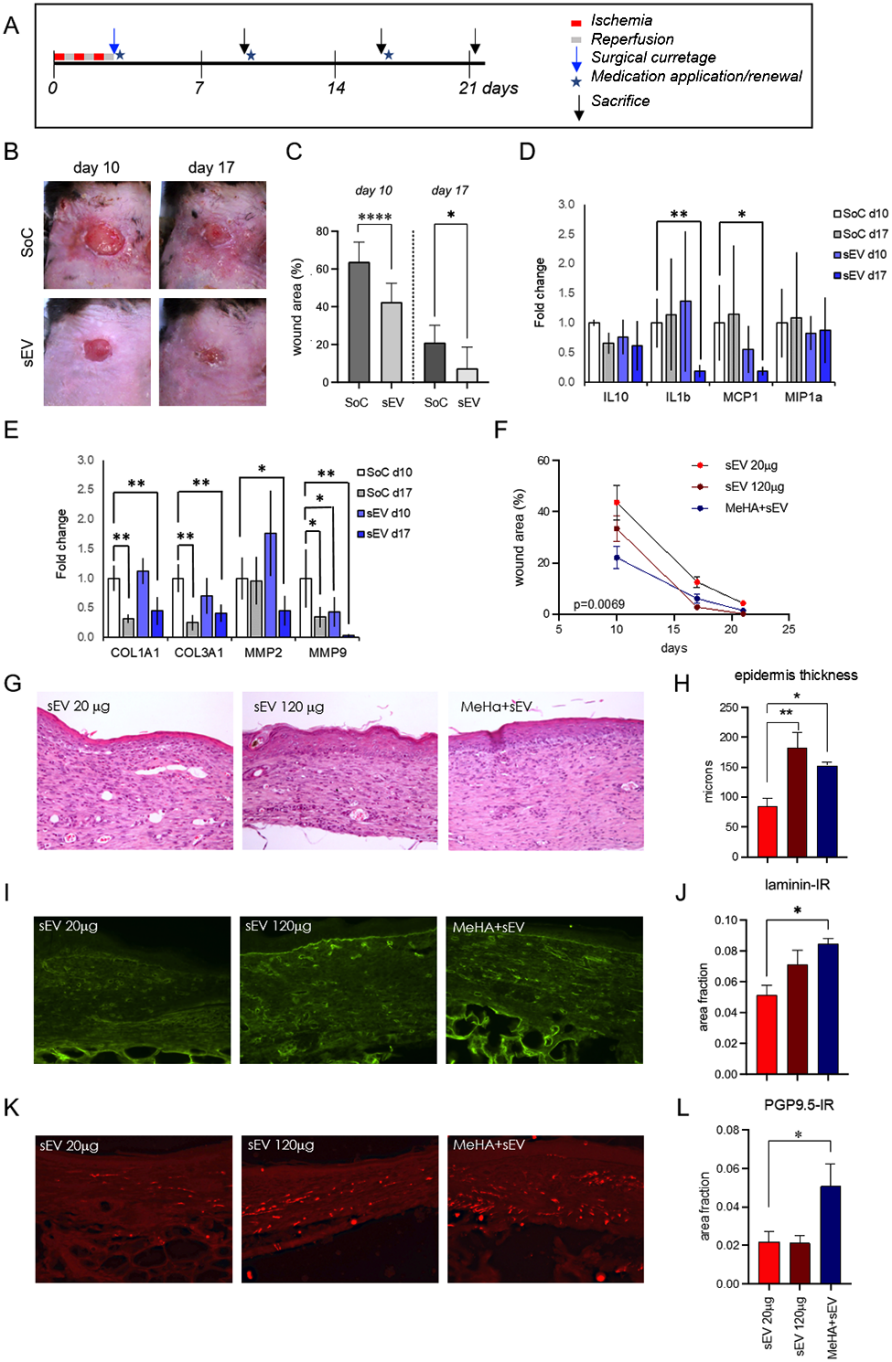
In vivo experiments on pressure ulcer model. **(A)** Schedule of the experiment. **(B)** Representative photos of the ulcers at different post-lesion times in standard of care (SoC) and MSC-sEVs treated (Exo) db/db mice. **(C)** Ulcer areas expressed as a percentage of the skin lesion area at day 0. **(D)** Cytokines expression profile. (E) Extracellular matrix proteins and remodeling enzymes expression profile. **(F)** Wound repair in ulcers medicated with 120 µg or 20 µg of MSC-sEVs and MeHA patches previously loaded with 120 µg MSC-sEVs. **(G)** Representative micrographs of hematoxylin-eosin staining in skin biopsy. **(H)** Morphometric evaluation of epidermal thickness. **(I)** Representative micrographs of laminin-IR in skin biopsy. **(J)** Morphometric evaluation of laminin-positive percentage area. **(K)** Representative micrographs of PGP 9.5-IR fibers in skin biopsy. **(L)** Morphometric evaluation of PGP 9.5-positive percentage area. *p < 0.05, **p < 0.01, ****p < 0.0001

In vivo experiments were performed with the authors’ extensively characterized pressure ulcer model [33, 34]. The model consists in the induction of three cycles of hypoxia/ischemia on the back of db/db diabetic mice (Fig. 6A). MSC-sEVs efficacy on wound healing compared to the standard of care (SoC, sterile TNT gauze moistened with saline solution) was firstly tested. MSC-sEV-treated wounds and surrounding skin appeared much less inflamed compared to SoC at visual inspection (Fig. 6B) and repaired more rapidly (Fig. 6C). The gene expression profile revealed that MSC-sEVs had a modulatory effect on some inflammatory factors and remodeling enzymes involved in wound healing (Fig. 6D and F). Compared with SoC, MSC-sEVs significantly reduced the expression of the cytokines IL1b and MCP1 (Fig. 6D), and metalloproteinase MMP2 and MMP9 (Fig. 6E). In a second experiment, different doses of soluble MSC-sEVs and the MeHA printed patches loaded with MSC-sEVs were tested. A faster wound repair in mice medicated with high (120 µg) compared to lower (20 µg) dose of MSC-sEVs was observed, and the repair was further improved by loading MSC-sEVs (120 µg) in MeHA patches (Fig. 6F). Biopsies in repaired skin were analyzed to evaluate re-epithelization by measuring the epidermal thickness in hematoxylin-eosin-stained sections (Fig. 6G H), angiogenesis by evaluating the laminin-IR area in the derma (Fig. 6I J), and terminal innervation by evaluating the PGP9.5-IR area in the derma (Fig. 6K L). All these indices of skin repair were improved in mice medicated with MSC-sEVs loaded into MeHA patches.

## Discussion

Diabetic foot wounds represent an overall major social, economic, and public health problem and need advanced treatment. HA-based hydrogel medications have shown beneficial effects as wound dressings, particularly due to their ability to ensure the sustained release of bioactive molecules, such as antibiotics, anti-inflammatory agents, growth factors, nanoparticles, or stem cells [35–39]. In the present study, sEVs were chosen as bioactive factors that replicate the therapeutic effect of MSCs on wound healing process while avoiding their shortcomings, mainly potential tumorigenicity, immune incompatibility, and frequent apoptosis [40, 41]. The most common method of sEVs administration is injection; nonetheless, the injected sEVs are rapidly cleared by body fluids, thus limiting their beneficial effect. Conversely, the healing of diabetic wounds requires a relatively long time. In the light of these considerations, in the present work the main goal was to develop bioactive and non-invasive wound dressings that can serve as controlled release carriers for MSC-sEVs to prolong their survival and biological activity in the diabetic wound area.

In this study, the sEVs were produced by MSCs previously seeded into handled bioreactors, with the aim to increase the cell expansion factor and to recreate in vitro a growth environment more similar to the in vivo condition [27]. Furthermore, 3D growth systems influence MSC phenotype by increasing their secretory activity, and the relative secretome by promoting wound healing ability [42]. The sEVs isolated from serum-free CM of the 3D cultured MSCs were analyzed for dimension, size distribution, morphology and expression of specific markers. In particular, the particle size distribution was between 50 and 190 nm, with particle diameter mean of 130 nm and mode of 92 nm. The semi-quantitative exosome antibody array revealed positivity for ALIX, FLOT1, ANXA5, CD63 and CD81, weak positivity for ICAM, TSG101, and EpCAM, and negativity for the cellular protein contamination marker, GM130. Therefore, the sEVs express at least one protein from each category of transmembrane and intracellular proteins as required by the Minimal Information for Studies of Extracellular Vesicles (MISEV) [29]. Moreover, flow cytometry analysis confirmed the sEVs positivity for CD63 and CD81 as well as the positivity for CD90 and CD73, validating the origin from MSCs [28].

Before loading onto the MeHA-based patches, a series of in vitro experiments were performed to assess whether and at what concentrations the MSC-sEVs affect cellular functions of endothelial cells and dermal fibroblasts, which play an important role in the healing process of the skin [43]. Both cell populations internalized MSC-sEVs. Furthermore, the results highlighted that the exposure to sEVs enhanced both the proliferation and migration abilities of endothelial cells and fibroblasts, consistently with other reports [25, 44, 45]. Cell proliferation and migration are two key processes for successful wound healing, being required for the formation of granulation tissue that supports re-epithelialization [46]. Specifically, wound healing begins when fibroblasts proliferate and migrate into the wound site producing new ECM components, such as COL3, ELN, and GAGs, which are necessary for cell migration [47]. Gene expression revealed that the MSC-sEVs significantly modulated COL3A1 and ELN mRNA production, whereas they had little effect on the expression of COL1A1, in agreement with literature [48, 49]. MSC-sEVs had pro-angiogenic effects, as demonstrated by the tube formation assay and the increase in mRNA expression of the endothelial cell markers CD31, KDR and VWF. CD31 (alias platelet endothelial cell adhesion molecule-1) maintains endothelial cell junctional integrity [50]. KDR is the main receptor responsible for binding vascular endothelial growth factor (VEGF) and its expression correlates with an increase in endothelial cells proliferation, migration, and survival [51]. Lastly, the glycoprotein VWF modulating blood vessel formation regulates angiogenesis [52].

In regenerative medicine, hydrogels have been widely employed as a matrix cell and morphogen carriers to the site of injury. Recently, hydrogels have been also employed as bioinks so that the spatial organization of the printed cells and growth factors mimics that of the target tissue. In this case, the hydrogel ink serves as an extracellular adhesive to maintain the dimensional stability and mechanical strength. Additionally, hydrogels can offer ligands for certain interactions between the ECM and cell surface receptors that guide cellular processes like adhesion, migration, mitosis, differentiation, maturation, and protein production [53]. Among the hydrogels, HA and its derivatives like MeHA, have offered a wide range of possibilities in soft tissue engineering due the versatility of HA processing and its unique biological interaction with cells, which have made it an important building block for the development of new bio-functional materials [9]. Particularly, in the present study, an innovative smart patch was designed by 3D bioprinting of MeHA, which was later functionalized by MSC-sEVs.

When tested in a well-established diabetic mouse pressure ulcer model [33, 34], the efficacy of MSC-sEVs was further improved by the MeHA patch, which provided a constant release of sEVs over 7 days. At best of authors’ knowledge, this is the first study proving efficacy of MSC-sEVs in the pressure model of skin lesion, being full-thickness excisional wounds the most studied model [54]. However, the full-thickness excisional model does not include the ischemic insult, which is a key component in diabetic foot lesion [55], and the repair process is different from both a cellular and molecular point of view [34]. The skin lesions treated with MSC-sEVs appeared less inflamed and with improved wound healing rate when compared to SoC. We then investigated molecular and cellular events in the skin at a single time point, corresponding to healed wound. The gene expression profile revealed a modulatory effect in the expression of some inflammatory factors and remodeling enzymes involved in wound healing. Compared with SoC, MSC-sEVs significantly reduced the expression of the cytokines IL1b and MCP1, and metalloproteinase MMP2 and MMP9. As previously discovered by the authors, the gene expression profile of diabetic dermal tissue showed a dysregulation in inflammatory factors and metalloproteinase [56]. In diabetic wounds, high levels of cytokines including IL1b and MCP1 sustain the pro-inflammatory macrophage phenotype, which causes a significant delay in wound healing [57]. In normal healing, a large transient increase in MCP1 is followed by a return to physiological level within the first few days after injury. In diabetic wounds, conversely, a sustained high level of MCP1 is present until late phases of the repair process, resulting in elevated numbers of macrophages. Furthermore, persistent hyperglycemia stimulates endothelial cells to overproduce MCP1 exacerbating chronic wound healing [58]. Poor healing of diabetic ulcers also depends on high expression of MMPs including MMP2 and MMP9 that degrade ECM components and cleave growth factors essential for wound healing [59]. In particular, MMP9 selectively degrade the growth factors among witch VEGF that promote angiogenesis and wound healing [60]. MMP2 cleaves laminin to stimulate the migration of keratinocytes, and then the re-epithelialization [61]. Inhibition of these MMPs has been observed to accelerate the healing of diabetic ulcers [62], and the treatment with MSCs was shown to significantly reduce MMP9 expression in a diabetic animal model [63]. Diabetic mice treated with MSC-sEVs showed a persistent lower expression of MMP9, whereas MMP2 was upregulated in the early and downregulated in the last stage. The accelerated healing observed in mice treated with MSC-sEVs could be due to a modulation effect on the gelatinases, typically overexpressed in diabetic wounds, as the expression of collagen I and III does not undergo changes compared to the SoC. Furthermore, epithelization, angiogenesis, and innervation at wound closure were improved by MSC-sEVs or MSC-sEVs loaded on MeHA patches. MSC-sEVs-loaded MeHA patches are more effective also in favoring the sprouting of nerve fibers and innervation restoring in repaired skin. This is a remarkable biological effect, being small fiber differentiation reported in diabetic skin, supporting the classification of purely neuropathic and neuroischemic diabetic foot ulcers [64].

## Conclusions

In the present study, MSC-sEVs were loaded into MeHA patches to achieve positive synergistic effects on cutaneous wound healing. Notably, culturing MSCs in bioreactor maximized the production of MSC-sEVs that met the standards of MISEV. The efficacy of MSC-sEVs on human endothelial cells and dermal fibroblasts was first established in vitro by proliferation and migration assays and gene expression profiling. Hence, the MeHA patch was developed for the encapsulation, protection and release of MSC-sEVs in an in vivo pressure ulcer model. Wound closure in diabetic mouse was improved by MSC-sEVs and when encapsulated in MeHA patches, as demonstrate by epithelization, angiogenesis, and innervation assays. In conclusion, loading active cellular components such as MSC-sEVs into biomaterials represents a smart strategy to maintain the translational potential of mesenchymal cell while improving regulatory and economic impact.

## Methods

### MeHA synthesis

High molecular weight hyaluronic acid sodium salt (HAs, M_w_ = 1.5–1.8 10^6^ Da from *Streptococcus equi*, Sigma Aldrich, Milan, Italy) was modified to graft photoactive polymerizable groups by reacting with methacrylic anhydride (Me, Sigma Aldrich Milan, Italy) as previously described [31, 65, 66]. Briefly, 1 g of HAs was dissolved in 10 mL of pure water (H_2_O, Carlo Erba, Cornaredo, Italy) and stirred at room temperature (RT) for complete dissolution. MeHA was obtained by reacting the primary hydroxyl groups (-OH) with Me at 4 °C, keeping the pH between 8 and 9 using a sodium hydroxide solution (NaOH, Sigma Aldrich, Milan, Italy). An excess of 30 mol% ME per (-OH) was used. The reaction was carried out for 12 h and it was stopped by precipitating MeHA into cold anhydrous ethyl alcohol (EtOH, Sigma Aldrich, Milan, Italy). The supernatant was recovered by vacuum filtration. The isolated MeHA polymer was solubilized in pure water then dialyzed against distilled water for 5 days and freeze-dried (LaboGene’s CoolSafe 55 − 4 PRO, Bjarkesvej, Denmark).

### ^1^ H nuclear magnetic resonance (NMR)

^1^ H Nuclear magnetic resonance (NMR, Bruker AVIII 400HD, Fällanden, Swiss) was employed to assess the success of the functionalization reaction. MeHA (5 mg/mL) was completely dissolved in deuterium oxide (D_2_O) by using a vortex mixer, and it was transferred into NMR tubes. The data were collected at a frequency of 400 MHz. Phase and baseline corrections were applied before obtaining the areas (integrals) of purely absorptive peaks. The presence of methacrylate moieties on the HAs backbone was confirmed by the peaks at 5.7 and 6.2 ppm.

### 3D printing of MeHA

Freeze-dried MeHA was dissolved in *di*H_2_O at a concentration of 4% (*w*/*v*) containing 0.1% (*w*/*v*) 2-hydroxy-4′-(2-hydroxyethoxy)-2-methylpropiophenone (Irgacure 2959, Sigma Aldrich, Milan, Italy), as previously reported [31]. Briefly, 3D printing was performed on “Rokit Invivo 4D2” (Rokit Healthcare Inc., Seoul, Korea) using 1.80 firmware. The input printing model was sliced with a grid pattern using New Creator K 1.57.70. The printing speed was set at 6 mm/s. The dispenser temperature was set at 15 °C, while the bed was set at 0 °C. A needle of 0.6 mm, a layer thickness of 0.4 mm and a fill density of 50% were used to build porous patches measuring 50 mm × 50 mm × 3 mm. During printing, UV light (λ: 365 nm) was used to crosslink the biomaterial ink enhancing the mechanical properties and avoiding the collapse of the structures. After printing, 3D porous structures were also post-crosslinked in a UV cabinet (Analytik Jena UVP crosslinker, CL-1000, λ: 365 nm) for 10 min. Finally, the patches were freeze dried for 24 h and stored at -80 °C before using.

### Dynamic mechanical analysis

A-Q800 (TA-Instrument, New Castle, DE, USA) was employed to assess the mechanical properties of 3D printed structures. The frequency was varied between 0.5 and 5 Hz, using an amplitude of 100 µm in compression, a preload of 0.001 N and a force track of 125%. The tests were performed in a closed chamber in a wet state at room temperature. Elastic modulus (E’) is reported as mean value ± SD, n = 5.

### Swelling behavior

Dried 3D patches were weighted (w_0_) scaffolds and placed in 5 mL of sterile medium that was supplemented with antibiotics and allowed to swell under physiological conditions for up to 5 h (pH = 7.4, T = 37 °C). The swollen hydrogels were then removed at specific time intervals, immediately blotted on filter paper to remove the superficially absorbed water, the weight was measured (w_t_), and the samples were then put back into the solution. Equation (1) was used to calculate the swelling ratio (Q):

1 $$Q{\text{ }} = {\text{ }}\left( {{w_t} - {w_0}} \right)/{w_0}$$

Results have been reported in Fig. 5G as mean value ± SD, n = 5.

### Scanning electron microscopy

The 3D printed MeHA patch was observed by scanning electron microscopy (SEM, FEI Quanta 200 FEG, Hillsboro, OR, United States). Before the analysis, 3D patches were prepared as described before, frozen, lyophilized for 48 h. The lyophilized structure was coated with an ultrathin layer of Au/Pt by using an ion sputter and then observed by SEM.

### Incorporation and release of MSC-sEVs from MeHA dressing

The 3D printed MeHA patches were placed into a 24-well tissue culture plate and loaded with 20 or 120 µg of MSC-sEVs resuspended in 100 µL PBS. Control condition (0 µg) was MeHA patches loaded with 100 µL PBS. The release profile was evaluated by placing the loaded dressing in a 24-well plate containing serum-free culture medium for up to 11 days. Then, samples were withdrawn at selected time points and stored at -80 °C until all samples were collected. The release of sEVs were quantified using the BCA Protein Assay Kit (Thermo Fisher Scientific) according to the manufacturer’s instructions. Data reported as mean value ± SD, n = 3.

### MSCs culture in bioreactor

Commercially available MSCs (Human Adipose-derived Mesenchymal Stem Cells; ScienCell Research Laboratories, Inc., CA, USA) were seeded at a density of 5 × 10^5^ cells into handheld 3D bioreactors (VITVO®; Rigenerand Srl, Medolla, MO, Italy) [27] up to 14 days. The bioreactor is a closed system with two optical transparent membranes that allow gas exchange. Internally, a 400 μm matrix of biocompatible polyester separates two chambers. Each chamber has a port acting as input or output depending on the media flow. By input port, liquid can enter the first chamber, pass through the 3D matrix, fill the second chamber, and exit through the outlet port. Thus, the 3D matrix works as a filter that retains cell and as substrate for cell growth. MSCs at passage 3 were resuspended in 1.4 mL of culture medium (DMEM, high glucose, no glutamine, no phenol red; Thermo Fisher Scientific, Waltham, MA, USA) completed with exosome-depleted fetal bovine serum (Capricorn Scientific GmbH, Ebsdorfergrund, Germany) and injected into bioreactors by a syringe through one of the two ports provided. Then, bioreactors were placed in an incubator set at 37 °C and 5% CO_2_. Fresh medium (1.5 mL) was injected through both ports every 48 h and an equal volume of CM was collected. In 14 days, about 20 mL CM was collected for each bioreactor. At each harvest, CM was centrifuged at 300xg for 10 min at RT to eliminate dead cells, filtered with a 0.22 μm filter to remove cell debris.

### Calcein AM staining

The growth and viability of MSCs into bioreactors were detected by staining with 1 µM Calcein AM (Life Technologies, Chagrin Falls, OH, USA) for 20 min at 37 °C and 5% CO_2_. After removing the excess probe, a z-stack (z-step 2.5 μm) was acquired with a confocal microscopy (Nikon A1 confocal microscope, Nikon Corporation, Tokyo, Japan) equipped with a 10X objective.

### Cell flow cytometry

MSCs were incubated with the following fluorescent monoclonal mouse anti-human antibodies: CD44 FITC (BD Biosciences, San Jose, CA, USA); CD73 APC (eBioscienceTM, Thermo Fisher Scientific); CD90 PE (eBioscienceTM); CD105 PE (BD Biosciences); CD14 PE (eBioscienceTM); CD34 FITC (eBioscienceTM); CD45 APC (eBioscienceTM); HLA-DR FITC (eBioscienceTM). After two washes, fluorescent cells were detected with Attune NxT flow cytometer (Thermo Fisher Scientific). Data were analyzed using Attune NxT software (Thermo Fisher Scientific). Experiments were performed in triplicate.

### sEV isolation

The clarified CM was loaded onto Amicon Ultra-15 100 kDa centrifugal devices (Amicon Ultra-15 centrifugal ultrafilters with Ultracel-PL PLGC membrane, 100 kDa; Millipore, MA, USA), previously sterilized with 70% ethyl alcohol (Sigma-Aldrich, Saint Louis, MA, USA), and centrifuged at 2000xg for 20 min at 4 °C. The filtrate was washed with sterile PBS (Euroclone, Milan, Italy) through a further centrifugation at 2000xg for 20 min at + 4 °C. The hMSC-SEVs were finally recovered from the filtering unit, quantified by Pierce™ BCA protein assay kit (Thermo Fisher Scientific), and stored in small aliquot at -80 °C immediately.

### Nanoparticle tracking analysis

Nanoparticle tracking analysis (NTA) uses laser light scattering and Brownian motion to determine EVs size and concentration. Measurement of particle size and particle size distribution was performed with Nanosight NS300 (Malvern, UK) instrument equipped with a 488 nm laser. All samples were diluted in filtered PBS to a final volume of one mL. Ideal measurement concentrations were found by pre-testing the ideal particle per frame value (20–100 particles/frame). For each measurement, five 1-min videos were captured under temperature 25 °C and syringe pump speed 30. Data are represented as averaged finite track length adjustment (FTLA) concentration / size.

### Transmission electron microscopy (TEM)

For TEM acquisition, the protocol described elsewhere was follow [67]. Briefly, the sEVs were fixed in a 2% glutaraldehyde solution in phosphate buffer (ratio 1:1). The sEVs were then deposited, rinsed, and stained with heavy metal compounds onto a gridded slide according to the standard protocols. The slide was visualized with a TEM Zeiss EM 910 instrument (Zeiss, Oberkochen, Germany).

### Exosome antibody array

The immunoblotting analysis of sEV specific markers were performed using the commercial Exo-Check™ exosome antibody array (Systems Biosciences, USA) according to the manufacturer’s instructions. The array contains eight known sEV markers, including CD63, CD81, ALIX, FLOT1, ICAM1, EpCam, ANXA5, and TSG101; four controls, including two positive controls (HRP Detection), blank spot (background control) and GM130 cis-Golgi marker, which monitors for any cellular contamination. Briefly, 50 µg of MSC-sEVs was lysed and labeled for 30 min with constant mixing. The labeled samples were washed and blocked with the blocking buffer. Array membrane was incubated with labeled lysate/blocking buffer mixture at 4 °C overnight on a rocker. The next day, the membrane was washed and incubated with a detection buffer for 30 min at RT on a shaker. The membrane was washed and the chemiluminescence was developed with Clarity Western ECL substrate (Bio-Rad, USA). Membrane array was developed on the chemiluminescence imaging system (ChemiDoc, Bio-Rad). Experiments were performed in triplicate.

### sEV flow cytometry

sEVs were harvested with exosome-Human CD81 Flow Detection (from cell culture) (Thermo Fisher Scientific), according to the manufacturer’s instructions. In detail, 50 µL of sEV suspension was added to a tube containing 20 µL of CD81 magnetic beads, previously washed with 500 µL of Assay Buffer containing 0.1% bovine serum albumin (BSA, Sigma-Aldrich) in PBS, and incubated at 4 °C overnight under stirring (650 rpm/min). After incubation, the bead-bound sEVs were isolated with the MagnaRack magnetic separator (Thermo Fisher Scientific) and washed twice with of Assay Buffer. Isolated CD81-positive sEVs were then labeled with mouse anti-human CD63 PE (eBioscience™), CD81 PE (BD Biosciences), CD73 APC (eBioscience™), or CD90 PE (eBioscience™). After 1-h incubation at RT protected from light on an orbital shaker (1000 rpm/min), the bead-bound sEVs were washed twice and suspended in Assay Buffer. Two controls were performed: PBS (vehicle) and ultrafiltrated exosome-depleted medium were stained instead of sEVs. Data were collected with Attune NxT flow cytometer (Thermo Fisher Scientific) and analyzed using Attune NxT Software v2.5 (Thermo Fisher Scientific). Experiments were performed in triplicate and the data represent the average.

### sEV internalization

sEVs or PBS (negative control) were stained with PKH67 (PKH67 Green Fluorescent, Sigma-Aldrich) for 20 min at 37 °C, as previously described [18]. The excess unincorporated dye was removed from the labeled solutions by using Exosome Spin Columns (MW 3000) (Thermo Fisher Scientific), following the manufacturer’s instructions. Then, 1 × 10^4^ dermal fibroblasts/cm^2^ (ATCC, MA, USA) or 1 × 10^4^ endothelial cells/cm^2^ (HUVEC; ThermoFisher Scientific) were incubated with the labeled sEVs or PBS for 3, 6, and 24 h in DMEM (EuroClone) or in Medium 200 PRF (M200PRF, ThermoFisher Scientific) without supplements), respectively. After incubation, cells were washed, and the nuclei were stained with Hoechst 33,342 (ThermoFisher Scientific) for 10 min at RT. Finally, cells were fixed with 4% paraformaldehyde, mounted with ProLong™ Glass Antifade Mountant (Thermo Fisher Scientific), then observed with a laser scanning confocal microscopy system (Nikon A1 confocal microscope, Nikon Corporation, Tokyo, Japan) equipped with a 63X objective. Experiments were performed in triplicate.

### RNA isolation, cDNA synthesis and real-time PCR

Total RNA was isolated from MSC-sEVs-treated cells with the RNeasy Mini Kit (Qiagen, Hilden, Germany). Total RNA from skin biopsies with RNeasy Fibrous Tissue Mini Kit (Qiagen, Hilden, Germany). The RNA quality and concentration of the samples was measured with the NanoDrop™ 2000 (Thermo Fisher Scientific). For the first-strand cDNA synthesis, 500 ng of RNA were reverse-transcribed using the QuantiNova™ Reverse Transcription Kit (Qiagen) in a SimpliAmp™ Thermal Cycler (Thermo Fisher Scientific). Real-time PCR was carried out using the designed primers (Table S1 and Table S2) at a concentration of 700 nM and QuantiNova SYBR Green PCR (Qiagen) on a StepOnePlus™ Real-Time PCR System (Thermo Fisher Scientific). Data analysis was performed using the 2ΔΔCt method [68], and presented as mean fold change of six measurements.

### Animal experiments

Genetically diabetic male mice db/db (strain C57BL/KsJ-m+/+Leprdb, Charles River Laboratories -Calco-Lecco) were housed under standard conditions. Blood glucose was measured (Contour XT, Bayer, Basel, Switzerland), and only mice with blood glucose ≥ 250 mg/dL were included in the study. Under anesthesia (isoflurane 3% plus 2 L/min O_2_), pressure ulcers were induced in the dorsal back using two magnetic disks of 12 mm diameter (anisotropic ferrite) and a thickness of 5.0 mm, with an average weight of 2.4 g and 1000 G magnetic force (Algamagnetic, Italy), as previously described [33, 69]. Briefly, a skin fold was raised and placed between the two magnets to generate a compressive pressure of 50 mm Hg [34]. Three ischemia-reperfusion (I/R) cycles were used, each single I/R cycle consisting of a period of 12 h of magnet placement followed by a rest period of 12 h without magnet. A surgical wound curettage was performed on day 3 to remove the ischemic skin and eschar. After curettage, mice were randomly assigned to the treatment groups (N = 5 in each group). Topical application of the medications began after curettage, weekly renewed and dressed with Tegaderm (3 M Health Care; Tegaderm Roll, St Paul, MN, USA). After curettage and before each dressing renewal, both wounds were photographed and wound areas measured using Nis-Elements AR 3.2 software (Nikon Corporation, Tokyo).

### Histology, immunofluorescence and image analysis

Mice were sacrificed at 7, 14 or 21 days according to the experimental target. Skin samples were quickly dissected and fixed in 4% (v/v) paraformaldehyde solution and picric acid–saturated aqueous solution in 0.1 M Sörensen’s phosphate buffer (pH 7.4). Right wounds were embedded in paraffin, sectioned at 4 μm, and stained with hematoxylin and eosin (H&E). For immunofluorescence, left wounds were fixed as above for 24 h, washed for 48 h in 0.1 M phosphate buffer 5.0% sucrose and quickly frozen. Cryostat Sect. (14 μm thick, HM550 Microm, Bio-Optica) were incubated overnight with primary antibody anti-laminin (rabbit, 1:200 dilution; SIGMA Aldrich) and anti PGP-9.5 (rabbit, 1:350 dilution; Proteintech) at 4 °C in a humid chamber. After rinsing, sections were incubated with secondary antiserum Cy2 Donkey anti-Rabbit IgG (Jackson Immunoresearch), rinsed and mounted in glycerol containing 1,4-phenylendiamine (0.1 g/L). Immunofluorescence images were taken by a Nikon Eclipse E600 microscope equipped with the Q Imaging Retiga-2000RV digital CCD camera (Q Imaging, Surrey, BC, Canada) and a motorized z-axis stage. Analysis was performed using the Nis-Elements AR 3.2 software, by applying the same procedure to all images under comparison. The immunoreactive area was calculated as area/fraction (percentage of immunoreactivity over 400 × 300 μm area). For morphological analysis, five images and two levels/animal were sampled at the center of the repaired ulcer. All analyses were performed blindly. Epidermal thickness was determined at the equator of the lesioned area by H&E staining on histologic sections in the same area. The mean value of five measurements/section and three sections per animal was used for the statistical analysis.

### Statistical analysis

The results were expressed as mean ± SD and analyzed by GraphPad Prism software. One-way analysis of variance (ANOVA) and Student’s t-test were used to evaluate the statistical significance (*p* < 0.05).

### Electronic supplementary material

Below is the link to the electronic supplementary material.

Supplementary Material 1
