# Supplementary material for: Stem cell-derived small extracellular vesicles embedded into methacrylated hyaluronic acid wound dressings accelerate wound repair in a pressure model of diabetic ulcer
Source: J Nanobiotechnology. 2023 Dec 7;21:469. doi: 10.1186/s12951-023-02202-9 (PMC10702007; doi:10.1186/s12951-023-02202-9)
Supplement: Supplementary file 1 — Supplementary Material 1 [file 12951_2023_2202_MOESM1_ESM.docx]

Supporting Information

**Stem cell-derived small extracellular vesicles embedded into methacrylated hyaluronic acid wound dressings accelerate wound repair in a pressure model of diabetic ulcer**

*Letizia Ferroni*, Ugo D’Amora, Chiara Gardin, Sara Leo, Luca Dalla Paola, Elena Tremoli, Alessandro Giuliani, Laura Calzà, Luigi Ambrosio, Alfredo Ronca, Barbara Zavan**

*Characterization of hMSCs by immunofluorescence*

Before seeding in the bioreactors, hMSCs (Human Adipose-derived Mesenchymal Stem Cells; cat. #7510, ScienCell Research Laboratories, Inc., CA, USA) at passage 3 were tested for the expression of stem cell-specific markers by immunofluorescence. Briefly, cells seeded at a density of 1 x 10^4^/cm^2^ onto glass coverslip (13 mm diameter) were fixed in 4% paraformaldehyde solution in phosphate buffered saline (PBS; EuroClone, Italy) for 15 min, then permeabilized for 30 min at RT in 0.1% Triton X-100 (Sigma-Aldrich, Saint Louis, MA, USA) prepared in PBS. After three washing with PBS, the cells were incubated in 2% Bovine Serum Albumin (BSA; Sigma-Aldrich) solution in PBS for 1 h at RT. The cells were then incubated overnight at 4 °C with the following primary antibodies prepared in 2% BSA and 0.1% Triton X-100 in PBS: mouse monoclonal to CD44 (cat. #MA5-13890, ThermoFisher Scientific, Waltham, MA, USA) diluted 1:100; rabbit monoclonal to CD73 (cat. #ab133582, Abcam, Cambridge, UK) diluted 1:50; rabbit monoclonal to CD90 (cat. #ab133350, Abcam) diluted 1:50; mouse monoclonal to CD105 (cat. #MA5-11854, ThermoFisher Scientific); rabbit monoclonal to vimentin (cat. #ab92547, Abcam). Immunofluorescence staining was performed by incubating the cells for 1 h at RT with the following secondary antibodies prepared in 2% BSA and 0.1% Triton X-100 in PBS: Goat anti-Mouse IgG1 Alexa Fluor 488 (cat. #A-21121, ThermoFisher Scientific) diluted 1:1000; Goat anti-Rabbit IgG (H+L) Alexa Fluor 488 (cat. #A-11034, ThermoFisher Scientific) diluted 1:500; Goat anti-Rabbit IgG (H+L) Alexa Fluor 633 (cat. #A-21070, ThermoFisher Scientific) diluted 1:500. Alternatively, the cells were stained with Alexa Fluor™ 555 Phalloidin (cat. #A34055, ThermoFisher Scientific) diluted 1:50. Nuclear staining was performed with 2 μg/mL Hoechst H33342 (Sigma-Aldrich) solution for 15 min. The cells were coverslipped with a drop of ProLong™ Glass Antifade Mountant (Thermo Fisher Scientific), then observed with a laser scanning confocal microscopy system (Nikon A1 confocal microscope, Nikon Corporation, Tokyo, Japan) equipped with a 63X objective.


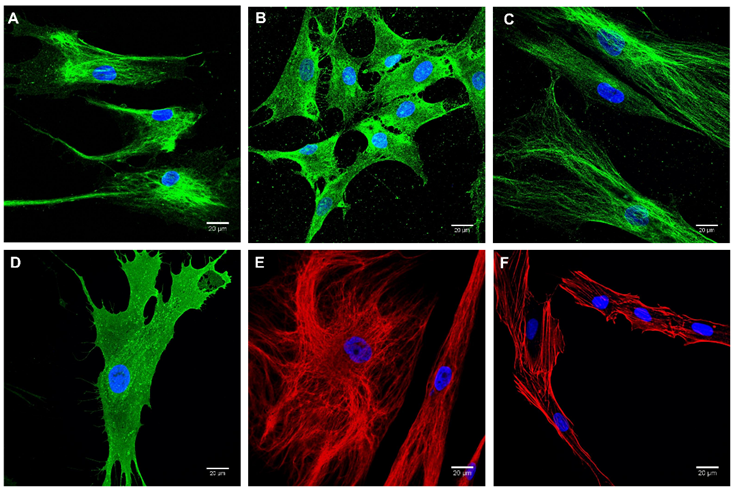


**Figure S1.** Human MSCs derived from adipose tissue at passage 3 adhered on plastic plates under standard culture conditions, and expressed specific surface markers. Confocal microscopy images of MSCs stained for (A) CD44, (B) CD73, (C) CD90, (D) CD105, (E) vimentin, and (F) actin filaments markers. Nuclei in blue. Scale bars 20 µm.

**Table S1.** Human primers list.

| **Gene** | **Primer forward** | **Primer reverse** | **Product length (bp)** |
| --- | --- | --- | --- |
| CD31 | TGTCTGAATCCAGGACGCTG | TGTGTGTTGGCTAAGGACCA | 195 |
| COL1A1 | TGAGCCAGCAGATCGAGA | ACCAGTCTCCATGTTGCAGA | 178 |
| COL3A1 | GGAAGAGATGGAAACCCTGGA | CACTTTCTCCTCTGTCACCAC | 172 |
| ELN | TGCACCCACCATCAACCTG | TGAAGTTCAGTGGACCGAGG | 186 |
| KDR | GGAGGAGGAGGAAGTATGTGACC | AACCATACCACTGTCCGTCTG | 184 |
| TFRC | TGTTTGTCATAGGGCAGTTGGAA | ACACCCGAACCAGGAATCTC | 222 |
| VWF | ACGTATGGTCTGTGTGGGATC | GACAAGACACTGCTCCTCCA | 159 |

CD31: cluster of differentiation 31; COL1A1: collagen type I alpha 1 chain; COL3A1collagen type III alpha 1 chain; ELN: elastin; KDR: kinase insert domain receptor; TFRC: transferrin receptor; VWF: von Willebrand factor.

**Table S2.** Mouse primers list.

| **Gene** | **Primer forward** | **Primer reverse** | **Product length (bp)** |
| --- | --- | --- | --- |
| COL1A1 | AGAAGCACGTCTGGTTTGGA | GTCCATGTAGGCTACGCTGT | 173 |
| COL3A1 | GTGGCAATGTAAAGAAGTCTCTGAAG | GGGTGCGATATCTATGATGGGTAG | 191 |
| GAPDH | TGGTGAAGGTCGGTGTGA | TTTGCCGTGAGTGGAGTCA | 158 |
| IL10 | AGTGGAGCAGGTGAAGAGTG | GGAGTCCAGCAGACTCAATACA | 159 |
| IL1b | TGCCACCTTTTGACAGTGATG | TGTGCTGCTGCGAGATTTGA | 135 |
| MCP1 | TGACCCCAAGAAGGAATGGG | ACCTTAGGGCAGATGCAGTT | 104 |
| MIP1a | TGCCAAGTAGCCACATCGAG | GAGATGGGGGTTGAGGAACG | 161 |
| MMP2 | ACAAGTGGTCCGCGTAAAGT | GTAAACAAGGCTTCATGGGGG | 189 |
| MMP9 | CGACTTTTGTGGTCTTCCCCA | TGCTTCTCTCCCATCATCTGG | 121 |

COL1A1: collagen type I alpha 1 chain; COL3A1collagen type III alpha 1 chain; GAPDH: glyceraldehyde-3-phosphate dehydrogenase; IL10: interleukin 10; IL1b: interleukin 1 beta; MCP1: mast cell protease 1; MIP1a: macrophage inflammatory protein 1-alpha; MMP2: matrix metallopeptidase 2; MMP9: matrix metallopeptidase 9.

*Optimization of structures design*

For the patches realization an accurate study about the design optimization has been conducted. It is widely known that hydrogel showed partial fibers coalescence during printing causing porosity collapse. Different infills have been tested from 40 to 70% to find the best balance in terms of pore dimension and interconnection. The 40% structure showed an open pore structure with pores too large to optimize the cell seeding (Figure S2 a). Rasing the infill at 60% the porosity starts to be closed due to the fibers collapse and the same could be seen for the 70% infill structure (Figure S2 c,d) For the 50% we have the best compromise in terms of cell diameter and interconnection (Figure S2 b). In this scenario structures with 50% infill showed an open porosity that allow cell seeding, nutrient exchange, wound draining and transpiration.


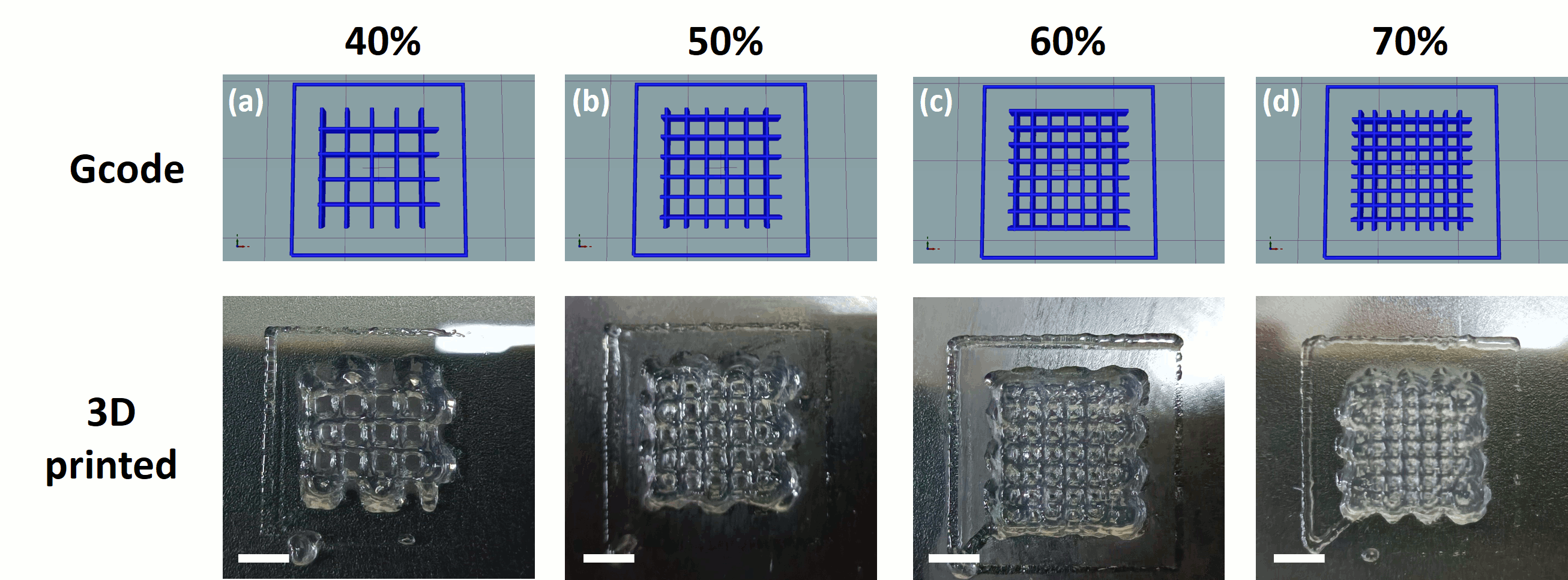


**Figure S2:** optimization of patches design in term of porosity and structure stability. In figure are reported structure with 40% (a), 50% (b), 60% 8c) and 70% (d) infill values.

*Degradation test*

The degradation behaviour of the patches was assessed by immersing the samples in physiological conditions (pH 7.4, 37 ◦C) for up to 15 days. At fixed time points, the patches were weighted. The remaining patches’ weight (W_r_) was calculated using Equation (1) and reported in Figure S3:

| *W_r_ (%) = w_t_/w_d1_ × 100%* | (1) |
| --- | --- |

where w_t_ and w_d1_ indicate the weight of the patches at the selected time intervals and at day 1, respectively. Data are reported as mean value ± SD, n=5

*
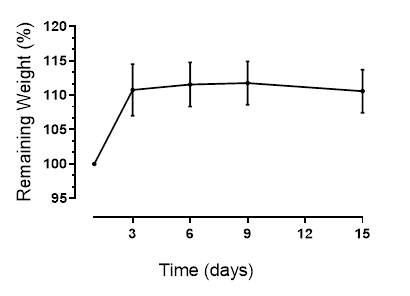
*

**Figure S3*:*** degradation profile of MeHA patches up to 15 days.
